# Root-mediated sex recognition in a dioecious tree

**DOI:** 10.1038/s41598-017-00894-2

**Published:** 2017-04-11

**Authors:** Tingfa Dong, Junyu Li, Yongmei Liao, Bin J. W. Chen, Xiao Xu

**Affiliations:** 1 Key Laboratory of Southwest China Wildlife Resources Conservation, Ministry of Education, and College of Life Sciences, China West Normal University, Nanchong, Sichuan 637009 China; 2 School of Urban-rural Planning and Landscape Architecture, Xuchang University, Xuchang, 461000 China; 3 College of Biology and the Environment, Nanjing Forestry University, Nanjing, 210037 China

## Abstract

Recent studies have demonstrated that plants can determine the identity of neighbouring roots (e.g., self and non-self, kin and non-kin), but whether they can discriminate by sex remains an open question. Here, we predict that dioecious plants can modulate their root performance in response to local root conditions related to sex. Female and male *Populus cathayana* cuttings were planted in a greenhouse in root-owner (one individual without a root neighbour) or root-sharer pairs (two individuals with roots neighbouring each other) with equal amounts of nutrients and space per plant in three combinations (females–females, males–males or females–males); root morphology, biomass and allocation were investigated. *P*. *cathayana* root-sharers altered their root growth in same-sex but not in different-sex combinations. Females enhanced root growth and allocation but decreased root proliferation (greater diameter with reduced branching and specific root length) in the presence of a female root neighbour, while males reduced root growth but increased root morphological proliferation in contact with another male. Therefore, the effect of a neighbour of the same sex differed from that of a neighbour of the opposite sex, which suggests that these plants can recognize the sexual identity of their neighbours.

## Introduction

Plant social interactive behaviour, especially as expressed in roots among plants growing with or without neighbours, has received increasing attention in the last two decades^1–5^, but these interactions remain relatively poorly understood in plants compared with animals^6–8^. Some studies show that plants can exhibit various behaviours (e.g., gene expression, growth, physiology, morphology and biomass allocation) in response to neighbours^4, 9–11^. These individual plastic responses can affect the composition and functioning of plant communities^12–16^ and plant ecology^17^ and evolution^18, 19^.

Several experimental lines of evidence have indicated that plants can respond to the presence of a root neighbour by altering root morphology and (or) allocating biomass to root growth^18, 20–22^ in herbaceous species, such as *Avena sativa* ^23^, *Brassica rapa* ^5^, *Glycine max* ^18^, and *Pisum sativum* ^11, 20^. A large comparative study of 20 species also reported that plants can reduce their overall root system size or adjust the horizontal and vertical placements of their roots in response to the presence of neighbours^24^. These results have indicated that root-level responses are related to neighbour identity in terms of species, kin versus non-kin or self versus non-self^8, 25^. However, to our knowledge, few studies have investigated the capacity of woody species to discriminate the identity of root neighbours (*but see* in *Cycas* ^26–28^).

Dioecious species play a significant role in maintaining the stability of the structure and function of ecosystems^29^, despite making up only 5–6% of total plant species^30^. In nature, biased sex ratios in plants are prevalent, and sex ratios in many dioecious populations change with environmental change^31^. An important research topic related to dioecious species is how sexes are spatially segregated to balance the sex ratio and to enhance population fitness^32^. Previous studies of dioecious species have focused on sex-related leaf responses to abiotic and biotic conditions. In *Populus* species, with respect to growth and physiology, leaves of females are generally more sensitive to environmental changes than those of males (e.g., water^33–35^, nutrient availability^36^ and competition^37, 38^). This sexual dimorphism is associated with a higher reproductive investment by females than males^32, 39, 40^. In addition to individual responses to changes in the amounts of environmental resources per plant, how roots respond to neighbour identity, even under equal amounts of resources per plant, may also be important for influencing population structure (e.g., sexual spatial segregation) in dioecious species^38, 41–43^. However, it is unclear whether dioecious plants can identify the sex of a root neighbour. If the effects of root interactions are sex-dependent, then root communication in dioecious plant species may influence the sex ratios and even the patterns of spatial sexual segregation.

Here, *Populus cathayana*, a widely distributed deciduous tree species in the northern hemisphere, was employed as a model tree species to investigate how females and males respond to the presence of same-sex or different-sex root neighbours. We studied sex-related root recognition in *P*. *cathayana*, focusing on the effects of root neighbours (owners or sharers) and their sex (intra-sex or inter-sex) by measuring root biomass and allocation, average root diameter, specific root length and root branching intensity in plants subjected to different treatments. Our objectives were to examine whether (1) the root growth of *P*. *cathayana* is affected by root neighbours; if so, whether (2) these responses are related to the sex of the neighbour; and whether (3) the root performances (biomass and morphological traits) of females and males are different in the presence of neighbouring roots.

## Results

### Biomass and allocation

**Figure 1:**
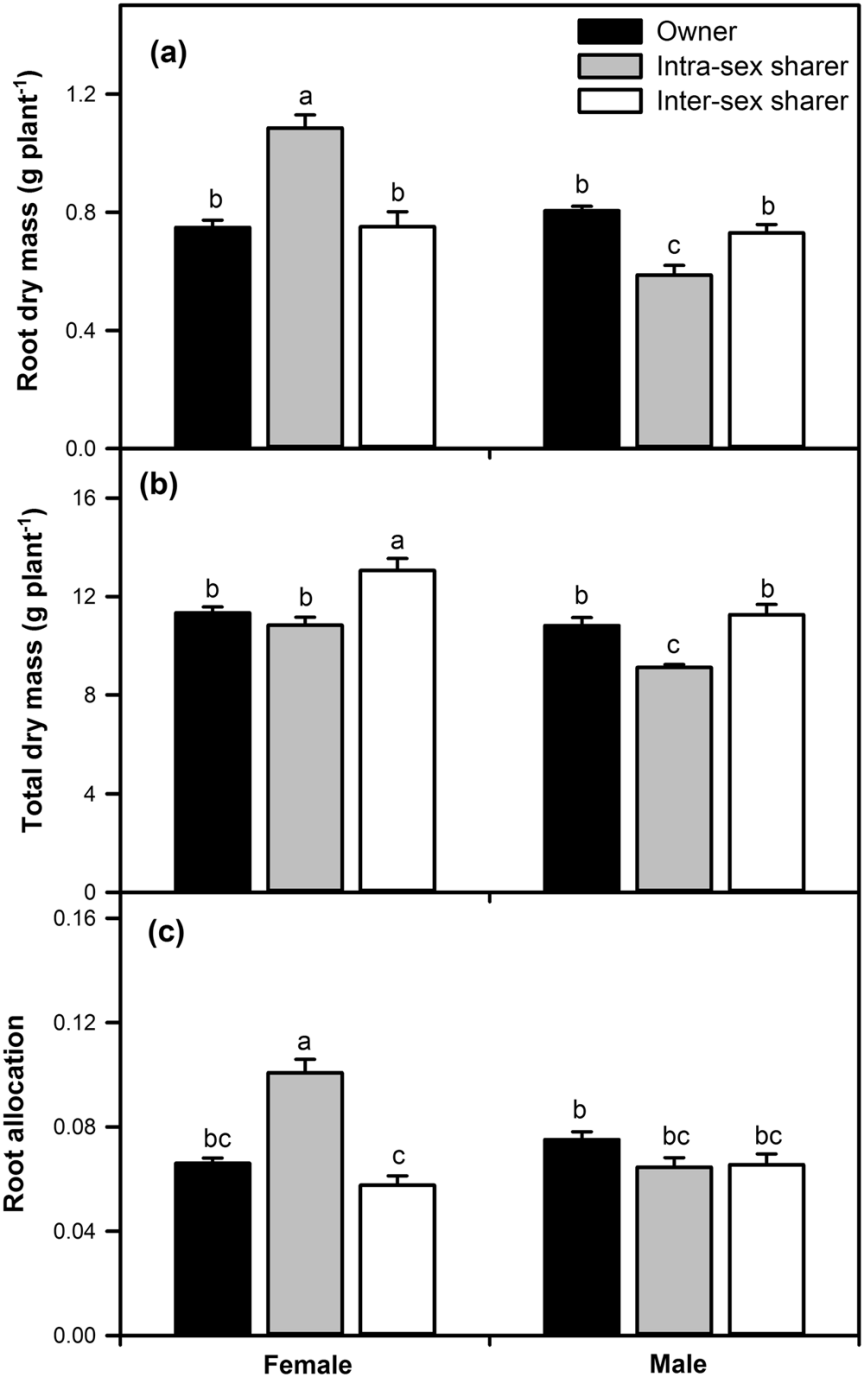
Effects of root neighbour combination treatments on root dry mass (**a**), total dry mass (**b**) and root mass fraction (ratio of root mass to total mass) (**c**) on female and male *Populus cathayana* cuttings grown in owner (no interplant roots; black bar) and sharing (with interplant roots from the same sex, grey bar; with interplant roots from the opposite sex, white bar) boxes. Each value is the mean ± SE (*n* = 10 in owner; *n* = 5 in intra-sex or inter-sex sharers). Bars with different letters are significantly different at the *P* < 0.05 level according to Tukey’s test.

Biomass accumulation and allocation of female and male *P*. *cathayana* individuals were significantly affected by root neighbour patterns (owner, intra-sex sharer and inter-sex sharer) (Table 1). Compared with owner individuals, females exhibited greater root dry mass (Fig. 1a) and root allocation (ratio of root mass to total mass, Fig. 1c) in the presence of an intra-sex sharing neighbour but had similar values of these traits in the presence of an inter-sex sharing neighbour. In males, the root dry mass and total dry mass of intra-sex sharing individuals decreased significantly, whereas the total dry mass and root allocation of an inter-sex sharer were not significantly different from those of an owner (Fig. 1).

**Table 1:** Summary of the effects of root neighbour patterns (owner, intra-sex sharer or inter-sex sharer), sex (female or male) and their interactions on *Populus cathayana* root biomass and allocation and root morphological traits according to linear mixed models.

	Root neighbour pattern (N)			Sex (S)			N × S		
	df	*F*-value	*P*-value	df	*F*-value	*P*-value	df	*F*-value	*P*-value
Root dry mass (g plant^−1^)	2, 40	7.312	**0.002**	1, 40	36.342	**<0.001**	2, 40	44.022	**<0.001**
Total dry mass (g plant^−1^)	2, 40	11.549	**<0.001**	1, 40	20.092	**<0.001**	2, 40	2.263	0.109
Root mass fraction	2, 40	16.074	**<0.001**	1, 40	6.870	**0.013**	2, 40	27.524	**<0.001**
Avg. root diameter (mm)	2, 40	25.419	**<0.001**	1, 40	0.000	0.994	2, 40	31.303	**<0.001**
Specific root length (m g^−1^)	2, 40	7.631	**<0.002**	1, 40	6.785	**0.014**	2, 40	34.647	**<0.001**
Root branching intensity	2, 40	3.402	**0.045**	1, 40	30.618	**<0.001**	2, 40	39.087	**<0.001**

Significant effects (*P* < 0.05) are shown in bold.

In addition to the type of root neighbour, sex also affected the root dry mass, root allocation, and total dry mass (Table 1). Although there were no significant differences in root dry mass, total dry mass and root allocation between the sexes in owner individuals, the values of these traits were significantly higher in female than in male intra-sex sharer individuals (Fig. 1). However, there were no significant differences in root dry mass and root allocation between female and male inter-sex sharer individuals. Finally, all of these values were significantly affected by the interaction of sex and neighbour (Table 1). Moreover, in intra-sex sharers in all treatments, the highest root dry mass and root allocation were observed in females, but the lowest root and total dry masses were found in males (Fig. 1).

### Root morphological traits

**Figure 2:**
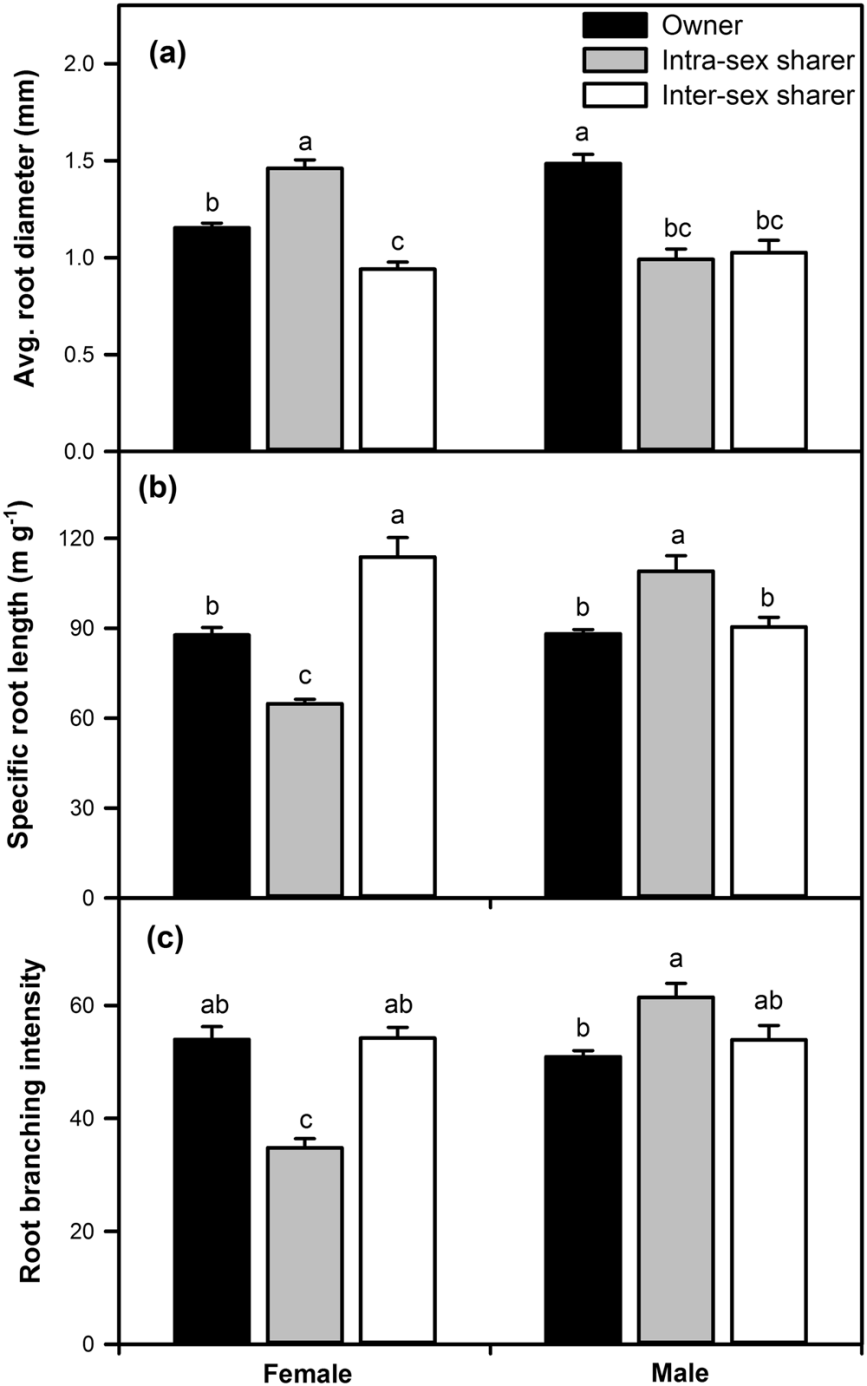
Effects of root neighbour combination treatments on average root diameter (**a**), specific root length (**b**) and root branching intensity (**c**) on female and male *Populus cathayana* cuttings grown in owner (no interplant roots; black bar) and sharing (with interplant roots from the same sex, grey bar; with interplant roots from the opposite sex, white bar) boxes. Each value is the mean ± SE (*n* = 10 in owner; *n* = 5 in intra-sex or inter-sex sharers). Bars with different letters are significantly different at the *P* < 0.05 level according to Tukey’s test.

The presence of neighbouring roots significantly affected root morphology (Table 1). Females in the intra-sex sharer treatment exhibited increased average root diameter but decreased specific root length (SRL) and root branching intensity (RBI), whereas the opposite results were observed in males (Fig. 2). In the presence of inter-sex root neighbours, average root diameters were lower in both females and males compared with owners (Fig. 2a), but SRL was higher in females (Fig. 2b). However, SRL and RBI in inter-sex male sharers were similar to those of the owners (Fig. 2b,c).

SRL and RBI were significantly affected by sex (Table 1). In root-owner individuals, SRL and RBI in females and males were similar, whereas these traits in female inter-sex sharing individuals were significantly lower than in males (Fig. 2b,c). Moreover, all root morphological traits were affected by the interaction of neighbour and sex (Table 1).

## Discussion

### Sex-related responses to root neighbours

Using a split-plot experimental design, our study showed that female and male *P*. *cathayana* exhibited variations in root biomass when exposed to sex-related neighbours sharing the same rooting space. Compared with plants growing without a neighbour sharing their rooting space, females had greater root dry mass and root allocation when grown with the same-sex neighbours but had similar root mass and root allocation when grown with male neighbours. In contrast, males grown with the same-sex neighbours produced lower root and total dry masses than those males grown alone (Figs 1 and 2). These results indicate that these plants can adjust the root biomass according to the sex of neighbouring plants by the interactions of the roots. Hence, our study confirmed that the dioecious tree *P*. *cathayana* can discriminate root neighbours based on sex. Previous studies have reported that plants could discriminate self/non-self neighbours at the species^1, 21^, kin^2, 6, 44, 45^ and genotype^46, 47^ levels, but no studies have shown that plants can be identified by sex. In forest ecosystems dominated by dioecious trees, in addition to kin/non-kin and self/non-self, the relationship between sexes is also an important part of the root interaction in underground root networks.

In addition to biomass, the root morphological traits of *P*. *cathayana* varied in response to root neighbours. Root architecture directly impacts nutrient acquisition and plant size because it affects the capacity of roots to compete for nutrients or space^48–51^. In this study, we found that the morphological root traits (e.g., root diameter, specific root length and root branching intensity) of *P*. *cathayana* were sensitive to the sex of root neighbours. A greater root diameter and lower SRL and RBI were observed in female individuals grown under same-sex sharing conditions, but a smaller root diameter and higher SRL and RBI were observed in females grown under different-sex sharing conditions. However, the opposite results were observed in males grown with same-sex root neighbours (Fig. 2). Since SRL and RBI are positively related to root absorption efficiency for nutrients^49–51^, our results suggested that females would decrease nutrient uptake when grown with female root-sharers but would increase nutrient uptake when grown with male root-sharers. The changes in plant nutrient uptake will change plant competitive capacity, resulting in different competition patterns among sex-related neighbours in a given nutrient environment^38^, which may result in variations of sex ratio and fitness^41, 42^.

### Mechanism of root recognition

A possible mechanism modulating self and non-self root interactions is communication through root chemical signalling, which has commonly been assumed in root and root-pair experiments^2, 5, 52^. Some studies have demonstrated that plant hormones (e.g., ethylene, strigolactone) and CO_2_ play key roles in plant responses to root neighbours by regulating the development of root architecture^53–55^, although no known study has shown that these substances can respond to sex-related root neighbours. In addition, plants can exude beneficial or harmful chemical substances (e.g., secondary metabolites) into their surroundings, and these substances can facilitate (or inhibit) the growth of neighbouring individuals^56^ or influence the relationship between the roots and soil microorganisms^57^. Several studies have found that plant root mass and morphology can respond to root exudates from siblings and unrelated species, as these chemicals can carry specific information regarding the genetic relatedness (*Arabidopsis thaliana* ^9^), population origin and species identity (*Deschampsia caespitosa* ^10^) of neighbours. These studies directly verify the hypothesis that root exudates can mediate neighbour recognition in plants, although which substances are responsible remains unclear. Previous studies also found that root exudates from *Populus* species are sensitive to changes to the surroundings^58^; however, further investigation into the role of root chemical signalling in the response of plants to the sex of a neighbour is needed.

In addition to the influence of root chemical signalling and root exudates, recent studies found that root volume seems to be another factor influencing the root neighbour effect in split-plot experiments^11, 59^. Within a split-plot experimental design, roots in a neighbour-present treatment (sharers) can occupy a larger space than those in a neighbour-absent treatment (owners), and sharer roots can produce more root mass in a soil substrate regardless of the presence of neighbours, although the amount of nutrients per plant is equal between the two treatments^11, 23, 59^. In our study, we cannot eliminate the potential effect of the volume of the containers used in the experiment, but plants grown in a Hoagland solution substrate may experience lower resistance in containers than in soil because roots grown on liquid substrate receive less horizontal support due to self-gravity, resulting in a more vertical root architecture than that found in a solid substrate; we did not observe lateral root tips touching the container wall during the experiment. Therefore, we hypothesize that our findings in *P*. *cathayana* are primarily caused by root interactions among individuals regardless of the rooting volume. This hypothesis is supported by the observation that if we consider only rooting-space-sharing treatments, root responses depended on the sex of the sharing individual and differed between female and male plants.

### Root neighbours affect sexual dimorphism

Sexual dimorphism in dioecious species is a result of different reproductive investments by females and males^40, 60–62^. The sex-related costs can result in different responses and capacities of acclimation to abiotic and biotic environmental stresses between females and males^33–35, 60–64^. These different sex-specific strategies influence sex-related interactions. Our previous study of *P*. *cathayana* reported differences between the sexes in ecophysiological traits under intra- and inter-sex competition^38^, but whether these differences were caused by roots remained unknown. In this study, we tested the role of roots in sexual interactions through a split-plot experiment and found that there were no significant differences in total dry mass between female and male *P*. *cathayana* cuttings when grown alone, but differences between sexes in this trait were significant when grown with same-sex or different-sex root neighbours. Compared with males, females made a greater biomass accumulation and root allocation when grown with same-sex neighbours. These sex-related differences in the response to the sex of root neighbours in our study may affect carbon accumulation and allocation, morphological growth, and the degree of dimorphism between female and male *P*. *cathayana* cuttings. According to Eppley^41^ and Chen *et al*.^37, 38^, sex-related differences in responses in growth and biomass allocation to root neighbours could directly change competitive effects between two sexes and result in biased sex ratios. This change, in turn, would influence the population structure of *P*. *cathayana* populations^33^.

In summary, this experiment showed that individuals of the dioecious plant *P*. *cathayana* altered their root biomass accumulation in same-sex but not different-sex combinations and exhibited opposite modifications of root biomass and morphology between females and males when grown with same-sex root neighbours. Our study demonstrated that *P*. *cathayana* can exhibit different responses to the presence of root neighbours according to the sex of the neighbour individuals. The results provide new evidence that these plants can discriminate sexual identity. However, additional studies involving the mechanism of regulating these sex-related responses to root neighbours in dioecious plants are required.

## Methods

### Plant material

*Populus cathayana* cuttings (including 30 males and 30 females) were collected from 60 trees (60 genotypes) sampled in different populations from Datong (35°56′ N, 101°35′ E) in Qinghai Province, China (*see* Xu *et al*.^33^ for a detailed description). Cuttings with similar sizes (approximately 10 cm) were planted in the experimental field of China West Normal University (30°48′ N, 106°03′ E; 276 m above sea level [a.s.l.]; the annual rainfall and annual temperature are 980–1150 mm and 15.6–17.4 °C, respectively, in northeast Sichuan, Southwest China. After sprouting and growing for approximately 2 months, female and male cuttings with similar root lengths (approximately 10 cm) and shoot heights (approximately 15 cm) were chosen and transplanted into glass boxes with running water and were allowed to adjust for two weeks.

### Experimental setup

The experiment, which started in the tenth week after cutting sprouting, was a completely randomized design with two factorial combinations of three neighbour effects (owners, intra-sex sharer and inter-sex sharer) and two sexes (female and male), and there were five replicate pairs per experimental treatment. Sixty cuttings were collected and planted in glass boxes filled with modified Hoagland solution according to Fodor *et al*.^65^. To block the sunlight, the top and circumference of each glass box were wrapped with black plastic bags, and all cuttings were kept in vertical positions with supporting bamboo poles. To assign individuals to “owner” or “sharer” treatments, we followed the method of Gersani *et al*.^18^, by which *P*. *cathayana* cuttings were planted in boxes that were undivided (40 cm × 20 cm × 30 cm for sharers) or were divided into two compartments with equal size (20 cm × 20 cm × 30 cm for owners). The roots of two individuals in a pair shared a glass box, and the distance between the stems was 20 cm. Pairs of ‘owner’ plants were kept with the same neighbour, and boxes with the experimental plants were completely randomly placed. The nutrient solution was renewed, and the position of each box was randomly shifted every 5 days. The space and resources per individual owner or sharer were held constant during the experiment. The cuttings were grown in a greenhouse under ambient light conditions at a temperature of 22–28 °C with 40–85% relative humidity.

### Measurements

At the end of the experiment (45 days after treatment), five cuttings from each treatment were harvested and separated into leaves, stems and roots. Their root morphological traits (e.g., total length and average diameter) were determined (WinRhizo, Regent Instruments, Inc., Québec, Canada), and the biomasses of all parts were then oven-dried (70 °C) to a constant weight and measured. In addition, the SRL and RBI (root length ratio between fine roots [diameter ≤2 mm] and coarse roots [diameter >2 mm]^10^) were also calculated.

### Statistical analysis

To test for root-mediated sex recognition, a general linear model was used to evaluate the effects of root neighbours (owners, intra-sex sharer and inter-sex sharer), sex (female and male) and their interactions on biomass and root morphological traits. Because the traits of females or males of owner pairs were not significantly different between same-sex and different-sex plants (*P* > 0.05 for all traits according to an independent sample t-test), we pooled the intra-sex and inter-sex owner pair data into either female or male “owner” sets. Differences among treatments were further investigated using Tukey’s comparisons at the *P* < 0.05 level. All analyses were performed using SPSS 16.0 for Windows (SPSS Inc., Chicago, IL, USA).
